# Supplementary material for: Gender differences in affective reactivity to COVID-related daily stressors in mood and anxiety disorders: A six-month intensive longitudinal study
Source: PLOS Ment Health. 2025 Dec 31;2(12):e0000328. doi: 10.1371/journal.pmen.0000328 (PMC12798290; doi:10.1371/journal.pmen.0000328)
Supplement: Attachment — Submitted filename: ResponseToReviewerComments_11.17.25.docx [file pmen.0000328.s002.docx]

**Response to Reviewers**

Journal Requirements:

**1. In the ethics statement in the Methods, you have specified that verbal consent was obtained. Please provide additional details regarding how this consent was documented and witnessed, and state whether this was approved by the IRB**

**Author’s response**: IRB approval was stated in the yellow highlighted section on page . Additionally, the following sentences were added on page 7, in the next paragraph to clarify how verbal consent was obtained

*“The study was approved by the Penn State institutional review board (IRB # 16212), and all participants provided informed consent. Data collection started 01/21 and ended 05/23. The study protocol was fully remote and approved by the Penn State IRB (#16212). Verbal consent was requested from participants. Potential participants were provided a description of the purpose of the study and study rationale, participant responsibilities, information to be collected, and risks and benefits of the study. Verbal consent approval was documented on the encrypted Penn State REDCap database.”*

**2. Please amend your detailed Financial Disclosure statement. This is published with the article. It must therefore be completed in full sentences and contain the exact wording you wish to be published.**

1. Please clarify all sources of funding (financial or material support) for your study. List the grants (with grant number) or organizations (with url) that supported your study, including funding received from your institution.

**Authors Response**: The following information is added to the title page. “T*his project was supported, in part, by funding from Penn State’s Social Science Research Institute (SSRI) to DM and EFHS. Content is the responsibility of the authors and does not represent the views of the SSRI. The funders had no role in study design, data collection and analysis, decision to publish, or preparation of the manuscript*.”

2. State what role the funders took in the study. If the funders had no role in your study, please state: “The funders had no role in study design, data collection and analysis, decision to publish, or preparation of the manuscript.”

**Authors Response**:	The following sentence has been added to the funding statement “The funders had no role in study design, data collection and analysis, decision to publish, or preparation of the manuscript.”

**3. Please send a completed 'Competing Interests' statement, including any COIs declared by your co-authors. If you have no competing interests to declare, please state "The authors have declared that no competing interests exist". Otherwise please declare all competing interests beginning with the statement "I have read the journal's policy and the authors of this manuscript have the following competing interests:"**

**Authors response**: "The authors have declared that no competing interests exist".

**4. Please provide separate figure files in .tif or .eps format.**

**Authors response**: We have provided figure file in .tif format.

For more information about figure files please see our guidelines: 
[https://journals.plos.org/mentalhealth/s/figures](https://urldefense.com/v3/__https:/journals.plos.org/mentalhealth/s/figures__;!!Ls64Rlj6!yFRXnQmhCc8G7CjhT3C2QTrmd-VWKDUjwnCIqC45i4YNxSGYnwq4gAnMYUydzFzK4oe7tIVAVVsEtNR5Sf-ggz5xx_E$) 
[https://journals.plos.org/mentalhealth/s/figures#loc-file-requirements](https://urldefense.com/v3/__https:/journals.plos.org/mentalhealth/s/figures*loc-file-requirements__;Iw!!Ls64Rlj6!yFRXnQmhCc8G7CjhT3C2QTrmd-VWKDUjwnCIqC45i4YNxSGYnwq4gAnMYUydzFzK4oe7tIVAVVsEtNR5Sf-gpZ0jrGs$)

**5. Please insert an Ethics Statement at the beginning of your Methods section, under a subheading 'Ethics Statement'. It must include:**

1) The name(s) of the Institutional Review Board(s) or Ethics Committee(s)

2) The approval number(s), or a statement that approval was granted by the named board(s)

3) (for human participants/donors) - A statement that formal consent was obtained (must state whether verbal/written) OR the reason consent was not obtained (e.g. anonymity). NOTE: If child participants, the statement must declare that formal consent was obtained from the parent/guardian.

**Authors Response:** The following paragraph has been inserted at the beginning of the Methods section as requested:

**“Ethics Statement**

*The study was approved by the Penn State institutional review board (IRB # 16212), and all participants provided informed consent. Data collection started 01/21 and ended 05/23. The study protocol was fully remote and approved by the Penn State IRB (#16212). Verbal consent was requested from participants. Potential participants were provided a description of the purpose of the study and study rationale, participant responsibilities, information to be collected, and risks and benefits of the study. Verbal consent approval was documented on the encrypted Penn State REDCap database.”*

**6. If the reviewer comments include a recommendation to cite specific previously published works, please review and evaluate these publications to determine whether they are relevant and should be cited. There is no requirement to cite these works unless the editor has indicated otherwise.** 


[Note: HTML markup is below. Please do not edit.]

Reviewers' comments:

**Reviewer's Responses to Questions**: Not applicable as no specific citations have been requested.

**Comments to the Author**

1. Does this manuscript meet PLOS Mental Health’s [publication criteria](https://urldefense.com/v3/__https:/track.editorialmanager.com/CL0/https:*2F*2Fjournals.plos.org*2Fmentalhealth*2Fs*2Fcriteria-for-publication/1/010f0198badc3727-83090e70-c433-4f53-a82e-a857d63c7e73-000000/juvN8J-jxJcfX8gqAP4fVVBIy1rNmCsIW5fJ-VNf-Nk=224__;JSUlJSU!!Ls64Rlj6!yFRXnQmhCc8G7CjhT3C2QTrmd-VWKDUjwnCIqC45i4YNxSGYnwq4gAnMYUydzFzK4oe7tIVAVVsEtNR5Sf-g4gGxfow$)? Is the manuscript technically sound, and do the data support the conclusions? The manuscript must describe methodologically and ethically rigorous research with conclusions that are appropriately drawn based on the data presented.

Reviewer #1: Yes

Reviewer #2: Yes

2. Has the statistical analysis been performed appropriately and rigorously?

Reviewer #1: Yes

Reviewer #2: Yes

3. Have the authors made all data underlying the findings in their manuscript fully available (please refer to the Data Availability Statement at the start of the manuscript PDF file)?

The [PLOS Data policy](https://urldefense.com/v3/__https:/track.editorialmanager.com/CL0/https:*2F*2Fjournals.plos.org*2Fmentalhealth*2Fs*2Fdata-availability/1/010f0198badc3727-83090e70-c433-4f53-a82e-a857d63c7e73-000000/rP7Qc__-QyBxzn2XVdlKBiJVDUfwrynoxtGmCQQa2N4=224__;JSUlJSU!!Ls64Rlj6!yFRXnQmhCc8G7CjhT3C2QTrmd-VWKDUjwnCIqC45i4YNxSGYnwq4gAnMYUydzFzK4oe7tIVAVVsEtNR5Sf-gUgw-ASE$) requires authors to make all data underlying the findings described in their manuscript fully available without restriction, with rare exception. The data should be provided as part of the manuscript or its supporting information, or deposited to a public repository. For example, in addition to summary statistics, the data points behind means, medians and variance measures should be available. If there are restrictions on publicly sharing data—e.g. participant privacy or use of data from a third party—those must be specified.

Reviewer #1: Yes

Reviewer #2: Yes

4. Is the manuscript presented in an intelligible fashion and written in standard English?

PLOS Mental Health does not copyedit accepted manuscripts, so the language in submitted articles must be clear, correct, and unambiguous. Any typographical or grammatical errors should be corrected at revision, so please note any specific errors here.

Reviewer #1: Yes

Reviewer #2: Yes

5. Review Comments to the Author

Please use the space provided to explain your answers to the questions above. You may also include additional comments for the author, including concerns about dual publication, research ethics, or publication ethics. (Please upload your review as an attachment if it exceeds 20,000 characters)

**Reviewer #1:**

Thank you for including me in this review. The authors present a paper examining gender differences in daily social and psychological stress and corresponding reactivity to stressful stimuli related to COVID-19. They show increased affective responsiveness for women vs. men in responding to COVID-19 related stressors, even as the frequency of these stressors remained constant between them.

The authors’ work offers an important, understudied angle on a still-timely topic. Overall, the paper is quite clear and coherently presented. However, it would strongly benefit from some re-organization; additional detail and context at a number of points; and a more thorough visual representation of the results. My comments are listed below by quote and page number, with major recommendations followed by minor suggestions.

Major recommendations:
**1. Page 3: “Women suffer from mood and anxiety disorders at rates higher than men…” While this opening introduces the topic of your paper, it feels like you are jumping into a specific, existing finding without any framing. I would recommend adding a sentence before this that generally frames why the work you’re doing is crucial, to grab the reader’s attention and contextualize this background further.**

***Authors’ response*:** We added the following opening sentence prior to “Women suffer from mood and anxiety disorders at rates higher than men..” 1^st^ sentence in the first paragraph, line 80.

*“Mood and anxiety disorders, impose a substantial public health burden worldwide, contributing markedly to disability, mortality, and lost productivity [1], a**nd have a bi-directional association with psychosocial and biological stress.”*

**2. Page 3: “differential occurrence and affective responsivity to daily stressors” – I would give an example of a past finding to contextualize this. Additionally, it’s not clear what is meant by “differential occurrence…to daily stressors” – please make sure that this is coherent.**

***Authors’ response*:** We added clarity to the statement from lines 86-88 which now reads as “As highlighted recently by Mazure and Husky, one potential source for this increasing discrepancy is women’s differential occurrence and affective responsivity to daily stressors [6].”

**3. Page 3, bottom: “Daily stressors elicit immediate emotional and physical effects on the day they occur…” – you contrast this with the longer-term effects of chronic stress, but it is not clear to me where an acute stressor (e.g. receiving a troubling email) might fit in. Would you argue that these daily stressors are also acute stressors, given their shorter time course? If not, I would recommend briefly addressing the distinction between daily stressors and acute stressors as they contribute/relate to chronic stress.**

***Authors’ response***: We thank the reviewer for the opportunity to clarify. Indeed, we are including acute stressors with the term “daily stressors.” We have further elaborated daily stressors in existing chronic and acute stressors frameworks (lines 99-102).

*“Recent research suggests that chronic stress can be conceptualized and assessed through the patterns of acute, short-term stressors and their responses. In this framework, daily stressors, typically acute stressors with a clear onset and resolution (i.e., stressor-free days), offer a valuable window into the dynamic nature of stress as it unfolds in everyday life [13].”*

**4. Page 4: “and both are causes of significant morbidity and mortality” – are daily stressors causes of these, or linked to higher morbidity and mortality rates by way of other factors? I would speak briefly to what kind of relationship these factors have to morbidity and mortality, and ensure that you cite a source for this point.**

***Authors’ response*:** We have edited the sentence to make it clearer as well as added a sentence linking positive affect to physical health outcomes from lines 114 -120.

*“Poor metabolic and cardiovascular outcomes occur at higher rates in those with mental illnesses [16, 17]. As daily stressors are increasingly linked to poor metabolic and cardiovascular outcomes, which in turn are linked to significant morbidity and mortality [18-20], it is worthwhile to understand the impact of response to daily stress in those with mental illness. The positive affect dynamics have been specifically linked to physical health outcomes. For example, evidence suggests that PA variability is predictive of health outcomes through distinct pathways from those linked to average PA levels [21].”*

**5. Page 4: “it is unknown whether affective response to stress will differ in men or women with these disorders” – this seems like one of the main questions of your paper, yet it feels buried in this paragraph with little lead-in. I would add a little more information contextualizing why this question is important and what we do know about it so far.**

***Authors’ response***: We added the following paragraph after the sentence that the reviewer has rightfully pointed out needs more context from line 124 -144.

*“The existing evidence of gender differences in daily stress processes in a non-clinical population suggests that women are likely to experience greater stress occurrences and heightened affective response to daily stressors (e.g., arguments or avoiding arguments) [22-24]. In addition, such gender differences may reflect underlying gender socialization and role-expectation processes: women’s greater involvement in unpaid labor and caregiving activities, and emotional/household management may both increase exposure to micro-stressors and amplify affective reactivity (for example, via higher emotional labor demands) [25, 26]. Establishing potential sex or gender differences in affective response to daily stress in those with mood and anxiety disorders is important for several reasons. First, epidemiological data show that many mood and anxiety disorders have markedly higher prevalence in women than in men [27, 28]. These prevalence differences raise the possibility that sex-specific psychophysiological or affective mechanisms contribute to risk for development of illness, and thus stress reactivity itself may show different patterns by sex in those with mood and anxiety disorders. Second, nonclinical and clinical studies have documented sex differences in stress physiology (e.g. cortisol responses, autonomic reactivity) and in emotional regulation more generally (e.g. women sometimes showing greater subjective negative affect or greater rumination under stress) [29, 30]. If such differences extend into clinical populations, they might help explain heterogeneity in symptom expression, treatment response, or resilience. Finally, from a translational or precision-psychiatry perspective, sex differences in affective stress response might point to differential targets for intervention (e.g. gender-tailored stress regulation strategies).”*

**6. Pages 4-5: “Moreover, the COVID-19 pandemic created novel stressful experiences across multiple domains.” – this appears to be the main focus of your paper. However, here it feels more like a minor point contextualizing your main question rather than the key circumstance under which the daily stressors you’re interested in arises. I would recommend leading a paragraph with this point, and perhaps citing a source (as well as for the sentences following it) discussing the impacts of the specific factors you’ve mentioned on people’s well-being. These may be self-evident but there’s also a lot of important research that can and should support your claims (e.g. Mukherjee & Pahan, 2021; Daly & Robinson, 2022, etc.).**

***Authors’ response***: The following paragraph was added after the sentence” Moreover, the COVID-19 pandemic created novel stressful experiences across multiple domains” (lines 148-161). The suggested Daly & Robinson, 2022, citation was also added. Mukherjee and Pahan, 2021 was not added since it focused more on molecular mechanisms and sex differences which is beyond the scope of the current paper.

*“The COVID-19 pandemic generated novel, multi-domain stressors—ranging from financial insecurity, social and relational disruptions, and job loss to illness risk and restrictions on mobility—which had pervasive impacts on mental health and daily well-being. Longitudinal evidence indicates that months characterized by greater COVID-related stressors were associated with heightened anxiety and depression and lower life satisfaction [31]. Similarly, data from the UK Household Longitudinal Study show significant within-person increases in psychological distress during pandemic waves, particularly among individuals experiencing caregiving and household burdens [32]. Daily-diary studies further demonstrate that on days with elevated pandemic worry, negative affect intensifies, and stress reactivity is magnified [33]. Together, these findings highlight the pandemic as a unique and multifaceted stress context that shapes daily emotional experiences and provides an essential backdrop for the current study’s investigation of affective stress responses. Interpersonally, social distancing created an isolated atmosphere when the need for emotional and social support was high”*

**7. Page 5: “the discrepancy in stress experienced by men and women may have been different, and gender differences may have been exacerbated.” – See above; there’s many great sources you could cite for these claims!**

***Authors’ response***: Thank you for pointing this out. We have addressed this claim along with the previous by adding the paragraph stated in the previous comment along with appropriate citations.

**8. Page 5: “In this study, we investigated the role of daily stressors…” Up to this point, you haven’t discussed how you are going to investigate the impact of daily stressors. Briefly discuss and justify why a daily diary paradigm is the method you chose to examine your questions.**

***Authors’ response***: Thank you for the reviewer’s comment. We have added the justification of using daily diary methods in the Introduction lines 174-178.

*“To address this aim, we utilized daily diary methods where participants repeatedly report their emotions, behaviors, and experiences once per day across consecutive days [34]. This method captures participants’ experiences in real time, allowing for assessing within-person and between-person variability of everyday experiences while reducing recall bias and enhancing ecological validity (Bolger et al., 2003).”*

**9. Page 5: Cite the PCARES and MDR databases.**

***Authors response***: We have cited the PCARES registry (Saunders et al., 2021;2023 and Gomaa et al., 2022). There is no citation for MDR and hence have replaced the word registry with repository -Mood Disorder Repository

**10. Page 6: “Participants were free to skip any questions that they preferred not to answer.” In the results section, you should report the extent to which questions were skipped. If many questions were skipped by many participants, be sure to discuss potential impacts on your results in the discussion section.**

***Authors’ response***: Thank you for the comment. For clarification, participants were given the option to select ‘prefer not to answer’ for each question. These responses were coded as missing. Specifically, one participant had missing data for education level, one for age, one for race, and one for PHQ score. This information is now clarified and included in the analytic plan (line 280-281).

*“Three participants were removed for multilevel modeling due to missingness in covariates (i.e., age, education level, race, and PHQ-9).”*

**11. Tables 1-2: While I appreciate your inclusion of the non-binary participant’s data, reporting information for n=1 in isolation could potentially enable participant identification. Be sure to verify that including this level of information for n=1 is permissible.**

***Authors’ response***: We agree with the reviewer’s comment that reporting data from one non-binary participant could potentially lead to participant identification. As this participant was also excluded from the analyses, we decided to remove their information from the reported results.

**12. Figure 1: If possible, please include individual data points on this figure (e.g. a scatterplot) so that we can see the distribution of affect and daily stressors per participant**

***Authors’ response***: As suggested, we have added individual data points in the updated Figure 1.

**13. Page 11: Ensure that the stats you provide are not repetitive to those in your table – you can discuss the statistics at the end of the Results section with reference to the table.**

**Authors’ response**: As suggested, we have condensed the descriptions of summary statistics in Table 2, emphasizing only the significant descriptive findings.

**14. Page 12: “The results show that the method is feasible and acceptable to participants.” – Is this something that participants reported in feedback to you, or is it inferred from completion rates? Please specify.**

***Authors’ response***: This was inferred from the completion rates. We therefore edited the sentence line 336-337 in the following way “*Based on the completion rates, the results suggest that the method is feasible and acceptable to mental health clinical populations.”*

**15. Page 12: “This may be because – in retrospect – we know that our study was conducted…” – in what way might the stress experiences have differed? For example, was COVID being reported less in the news? Were people around the participants getting it less often? Furthermore, how would this point explain the lack of observed gender differences in stressor occurrence? Would the progression of the pandemic and associated stressors have equalized the experience for women and men over time, for example?**

***Authors Response***: The reviewer’s point is well taken. In addition to our data being collected towards the tail end of the pandemic, while reviewing the COVID-19 pandemic and sex differences literature we found that the focus was more on the differential experience/response to COVID-19 stressors as opposed to the frequency of COVID-19 stressors. However, we added a paragraph to explain some potential reasons for the lack of differences as follows lines 350-360.

*“By this stage, many COVID-specific contextual stressors had attenuated: infection rates and hospitalizations had declined in many regions, vaccination campaigns had reduced perceived threat, media coverage became less central to everyday life, and people had adapted to new routines (e.g., remote work, masking, distancing) and the acute uncertainty that characterized the early pandemic [32, 50, 51]. Early in the pandemic, women disproportionately experienced stressors linked to caregiving demands, household responsibilities, and employment disruptions [52, 53]. As households, workplaces, and institutions adjusted over time, longitudinal evidence suggests some of these gendered differences in exposures and perceived burden decreased and adjusted over time, which may help explain why stressor occurrence was more similar across genders in our late-pandemic sample [54].”*

**16. Page 12: You discuss reasons for the expected gender differences in women quite thoroughly. However, in general, it would be important to further discuss/speculate why you may not have seen the expected gender differences in affective states as triggered by COVID-19 daily stressors.**

***Authors Response***: Thank you for the comment. We address differences in affective states in the following paragraph from lines 368 - 376. “*On the days when women encountered more COVID-related stressors than usual, they exhibited lower positive affect, suggesting heightened day-to-day affective reactivity to situational stress. Furthermore, women who experienced greater overall exposure to COVID-related stressors across the study period relative to those who did not, reported higher levels of negative affect, indicating more chronic stress-related distress. In contrast, these associations were not evident among men. These patterns together suggest that women may be both more emotionally reactive to daily stress and more vulnerable to the cumulative effects of stress exposure during the pandemic.*”

**17. Page 13, bottom: “The findings may have been different at depending on the type of COVID strain affecting individuals and/or restrictions imposed by the CDC.” – while this partly addresses my point above about time-dependent differences in the progression of the COVID-19 pandemic, I would again speculate further as to how each of the differences you mentioned might impact daily stressors (e.g. would perceived transmissibility, or decreased restrictions, be predicted to upweigh/downweigh responses to daily stressors respectively)?**

***Authors’ response*:** Thank you for the comment. This statement has been added to the limitations paragraph, lines 404-407.

*“During this later phase of the pandemic, widespread vaccine availability, improved treatment options, and societal adjustment and adaptation likely reduced the intensity of emotional responses compared to earlier, more uncertain phases of the pandemic.”*

**18. Page 14: “However, the strength of the daily diary study allowed for more comprehensive information on stressors, response and other variables of interest.” Increased frequency/sampling of responses does not necessarily mean that these responses are more comprehensive. Furthermore, I don’t know what you mean by “comprehensive information on…response” (which should also be plural). I would tighten this part up and be more specific in your justification.**

***Authors’ response***: We agree that the original sentence lacked clarity. We have revised it to provide more details from lines 411-414: “*However, compared with more intensive approaches, such as ecological momentary assessments, which involve brief but more frequent data collection, the daily diary approach includes a single assessment each day, allowing participants to provide extended and detailed reflections of their daily experiences*.”

**19. Page 14, end: I would recommend ending off with a more general/broadly applicable takeaway from your results, so that the reader knows the importance of your work to everyday life.**

***Authors’ response***: We agree and have changed the conclusion paragraph to the following

“*The findings demonstrate the importance of focusing on gender differences in stress response rather than the occurrence or frequency of COVID-19-related daily stressors. how Our findings gender highlights the gender differences in patterns of stress response, which in turn, could potentially inform interventions to address the impacts of the COVID-19 pandemic and overall daily stress in general.*”

**Minor suggestions:**
Title: You may consider rewording the title to indicate the direction of the relationship found between gender and reactivity to daily stressors; at the very least, it might be helpful to mention reactivity instead of just “processes”.

***Authors’ response***: Following the reviewer’s suggestion we reworded the title to “*Gender differences in affective reactivity to COVID-related daily stressors in mood and anxiety disorders: A six-month intensive longitudinal study*”

Abstract: “particularly during the COVID-19 pandemic” – add a comma after this.

Abstract: Add “an” before “increase in negative affect”.

Abstract: Add a comma between “novel” and “intensive”, as well as commas around “in turn”.

Page 7: “Unable to spend time with others” – I might recommend the phrasing “inability to spend time with others” instead, unless this is a verbatim quote of this event.

Pages 7-8: I would add in “emotions” right before “and negative” as these are long lists and the reader could lose track of what the categories are.

Page 9: Add “the” before “positive affect model” and “negative affect model”.

Page 10: Should “symptom” be plural?

Page 12: add “the” to “stress during COVID-19 pandemic”.

Page 12, end: “gender” should be plural.

***Authors’ response*:** The authors appreciate the attention to detail by the reviewer and have incorporated all the minor edits that have been suggested with track changes.

**Reviewer #2:**

The current study utilizes daily diary data collected during the COVID-19 pandemic to examine gender differences in daily stress processes related to the COVID-19 pandemic, including exposure and affective reactivity to COVID-19-related daily stressors. Strengths of the study include the intensive longitudinal design, the quality of the data, the assessment of daily COVID-19-related stressors, and the data analysis.


In addition to these strengths, there are issues that warrant further consideration to further strengthen the study’s contribution.

Introduction:

The Introduction is well-structured and grounded in research in contemporary research on stress and health.

**1. The Introduction would be further strengthened by elaborating more on potential explanations for the anticipated gender differences, including how exposure and reactivity to COVID-related stressors may be, in part, shaped by gender socialization, gendered expectations regarding division of labor, and gender differences in relationship monitoring and maintenance.**

**Further, given that the data was collected from January 2021 to May 2023, it would strengthen the contribution of the study to position the findings within a larger, less specific context, such as in times of uncertainty.**

***Authors’ response*:** We appreciate the feedback and have added the following tracked sentences within the introduction from lines 127-144.

“*In addition, such gender differences may reflect underlying gender socialization and role-expectation processes: women’s greater involvement in unpaid labor and caregiving activities, and emotional/household management may both increase exposure to micro-stressors and amplify affective reactivity (for example, via higher emotional labor demands) [22, 23]. Given the increased stress susceptibility caused by mood and anxiety disorders, it is unknown whether affective response to stress will differ in men and women with these disorders. Establishing potential sex or gender differences in affective response to daily stress in those with mood and anxiety disorders is important for several reasons. First, epidemiological data show that many mood and anxiety disorders have markedly higher prevalence in women than in men [24, 25]. These prevalence differences raise the possibility that sex-specific psychophysiological or affective mechanisms contribute to risk for development of illness, and thus stress reactivity itself may show different patterns by sex in those with mood and anxiety disorders. Second, nonclinical and clinical studies have documented sex differences in stress physiology (e.g. cortisol responses, autonomic reactivity) and in emotional regulation more generally (e.g. women sometimes showing greater subjective negative affect or greater rumination under stress) [26, 27]. If such differences extend into clinical populations, they might help explain heterogeneity in symptom expression, treatment response, or resilience. Finally, from a translational or precision-psychiatry perspective, sex differences in affective stress response might point to differential targets for intervention (e.g. gender-tailored stress regulation strategies)*.”

We also added from lines 163-166 the following sentence “*Moreover, during extended periods of societal uncertainty — such as the prolonged phase of the COVID-19 pandemic from early 2021 through 2023 —gendered pathways may be accentuated, as change in routine and differential distribution of household/work responsibilities lead to changes in interpersonal relationships.*” after the existing sentence “The uncertainty of when normalcy was going to return created anxiety and fear.”

Methods and Results:

**2. In addition to age, education level, and the PHQ-9, it would be helpful to explore, if available, additional covariates related to family structure, such as relationship status and number of children, which are likely to contribute to daily affect as well as COVID-19-related daily stressors.**

***Authors’ response***: We appreciate the reviewer’s insightful comment. As information on participant’s relationship status and family composition was not available in our dataset, we were unable to include these variables as covariates in the analyses. We have now noted this as a limitation and discussed its implications in the future directions section in lines 414-417.

*“Finally, we have not examined the impacts of contextual factors, particularly participant’s social relationship (e.g., family composition, married/cohabit status). Future research should examine how these factors shape daily stress and affective experiences among individuals with mental illness.”*

**3. The current study collected information on non-COVID-related stressors. It would be interesting to also consider gender differences in affective reactivity that consider how COVID-related stressors interact with non-COVID daily stressors. Considering how co-occurring daily stressors and COVID-related stressors contribute to affective well-being among people with mood and anxiety disorder would further strengthen the study’s contribution, particularly now that the significant daily stressors of the pandemic have ended by providing insights into gender differences in how everyday stressors interact with stressors associated with worry and uncertainty to shape affective responses within this under-studied population.**

***Authors’ response***: We appreciate the reviewer’s thoughtful comment. As suggested, we tested the interactions between COVID-19-related daily stressors and non-COVID-19-related daily stressors, as well as their three-way interactions with gender, on daily negative and positive affect. However, these interaction effects were not statistically significant, which may reflect limited statistical power or reduced variability in stressor exposure within our sample.

Discussion:

**4. The authors acknowledge several study limitations, including the limited racial and ethnic diversity in the sample. Given that the sample was also generally well-educated, this limitation should also be acknowledged and considered.**

***Authors’ response***: We added “and generally well-educated” in the limitations paragraph.

**5. Further, although the focus on COVID-19-related daily stressors is a novel contribution, given that we are several years past the most significant influences of the pandemic, it would strengthen the study to elaborate more on how the findings may inform interventions in response to other similar social contexts beyond the COVID-19 pandemic.**

***Authors’ response***: Thank you for the insightful comment. At the end of the discussion we have followed the reviewer’s suggestion and added the following paragraph in lines 389-400.

*“Importantly, the present findings also provide insights that extend beyond the COVID-19 pandemic. Periods of collective uncertainty—whether due to natural disasters, economic crises, or sociopolitical instability—share features with pandemic-related disruption, including chronic unpredictability, altered daily routines, and shifts in work and family roles [58, 59]. Understanding gendered patterns in stress reactivity under these conditions can therefore inform future mental health interventions aimed at bolstering resilience and adaptive coping in contexts of widespread uncertainty. For instance, interventions that target emotion regulation and social support among women, particularly those managing disproportionate caregiving or relational burdens, may buffer against the affective costs of acute and prolonged collective stress. Moreover, adaptive daily diary or ecological momentary assessment designs can be deployed in future crises to monitor fluctuations in affect and stress in real time, guiding personalized preventive and therapeutic responses [60].”*

**Additional Authors’ Note and Correction**:

As reviewing the analyses and manuscript during revision, we noticed the description of the interaction effects required correction. The interaction for positive affect was significant at the within-person level, whereas for negative affect, the significant interaction occurred at the between-person level. We have updated Figure 1 and Table 3, and refined the Discussion section to better reflect these results.
